# Detection of Hemiplegic Walking Using a Wearable Inertia Sensing Device

**DOI:** 10.3390/s18061736

**Published:** 2018-05-28

**Authors:** Junseok Lee, Sooji Park, Hangsik Shin

**Affiliations:** 1 Interdisciplinary Program in Biomedical Engineering, Chonnam National University, 77, Yongbong-ro, Buk-gu, Gwangju 61186, Korea; nszhang@naver.com; 2 Department of Biomedical Engineering, Chonnam National University, 50, Daehak-ro, Yeosu, Jeonnam 59626, Korea; susie.soo718@gmail.com

**Keywords:** accelerometer, hemiplegia, gait analysis, gyroscope, wearable

## Abstract

Hemiplegia is a symptom that is caused by reduced sensory and motor ability on one side of the body due to stroke-related neural defects. Muscular weakness and abnormal sensation that is induced by hemiplegia usually lead to motor impairments, such as difficulty in controlling the trunk, unstable balance, and poor walking ability. Therefore, most hemiplegia patients show defective and asymmetric gait pattern. The purpose of this study is to distinguish hemiplegic gait by extracting simple characteristics of acceleration signals that are caused by asymmetry during walking using a wearable system. The devised wearable system was equipped with a three-axis accelerometer and a three-axis gyroscope. We selected 165 candidate features without step detection. A random forest algorithm was used for the classification, and the forward search algorithm was also used for optimal feature selection. The developed system and algorithms were verified clinically in 15 normal subjects and 20 hemiplegia patients that were undergoing stroke treatment, and 26 subject’s data was used for training, including validation, and nine subject’s data used for test. As a result of test set, the accuracy, sensitivity, specificity and positive predictive value were 100.0%, with the two classification attributes of standard deviation of points perpendicular to the axis of line of identity of Poincaré plot of angular velocity around vertical axis and kurtosis of frequency of angular velocity around longitudinal axis.

## 1. Introduction

Stroke is the general term for cerebrovascular disease that is caused by cerebral artery occlusion or cerebral hemorrhage. Twenty percent of stroke patients recover completely from physical and mental impairment, 60% are affected by disorders of movement, speech, sensory, and cognitive systems, and the remaining 20% die [1]. Hemiplegia is a symptom that is caused by decreased sensory and motor ability on one side of the body due to stroke-related neurological defect. Therefore, a hemiplegic gait is characterized by muscular weakness and poor sensation inducing a movement disorder, such as difficulty in controlling the trunk or unstable balance [2]. Patients with hemiplegia have a tendency to move the center of gravity rapidly to the non-paralyzed side [3], to increase the walking speed and cadence per unit time or decrease the stride length and gait cycle to compensate for motor dysfunction [4]. There are three major differences in the hemiplegic gait when compared with the gait cycle of normal persons [5]. First, the duration of the stance phase is increased in both the paralyzed and non-paralyzed sides as compared with the normal gait. Second, the stance is more in the whole gait cycle than in the normal gait. Third, in the case of hemiplegic walking, the stance on the paralyzed side is noticeably shorter than on the paralyzed side, and the double support predominates the whole gait cycle. In paralysis and non-paralysis, there is a clear difference in the angle and position between the body segments during the stance, initial contact, swing phase, and toe-off.

In clinical practice, physical therapists use the manual muscle test (MMT), modified Ashworth scale (MAS), and range of motion (ROM) to evaluate motor function. The MMT divides the muscular strength into six levels according to the presence or absence of gravity, and the degree of resistance from the level without muscle movement to the level where it encounters a strong resistance of the examinee [6]. Each level is represented using a proposed alphabet or is scored from 0 to 5, as proposed by the Medical Research Council (MRC) [7]. The modified Ashworth scale (MAS) [8] is used to determine the degree of severity of rigidity at the six levels [9]. MMT, MAS, and ROM, which are mainly performed in clinical studies, involve naked eye observations of the degree of functional recovery. However, the disadvantage is that the detailed numerical result of the joint ROM cannot be recorded, and the subjective thought of the evaluator is reflected. In addition, since it does not consider the deterioration of the body according to aging, quantitative measurements and evaluation of the body movement have been actively studied. Representative methods include the measurement of the joint angle during walking using an electronic protractor, taking an action trace using a light emitter marker, and the use of a force plate [10,11,12]. The Vicon Motion Capture System (Oxford Metrics, Oxford, UK) is widely used in conventional gait analysis. The system records gait without constraining the body and yields accurate numerical records. However, disadvantages of the system include the need for large installation space and a high price [13]. Therefore, quantitative evaluation of walking ability in clinical practice is mainly performed in a place with specialized facilities. To address the limitations of quantitative evaluation, motion analysis using an accelerometer instead of the conventional three-dimensional (3D) motion analysis using a marker and video has been studied. The gait analysis system using the accelerometer is lightweight and portable, has no spatial limitations, and facilitates the analysis of spatial and temporal characteristics of walking. Studies analyzed the change in acceleration at the center of gravity with and without back pain [14]. Another study measured the acceleration in the fibular heads of hemiplegic patients and compared the acceleration at the paralyzed and non-paralyzed sides using the Brunnstrom stage, which measures the recovery phase of the arm [15]. Gait was measured using an accelerometer in 282 healthy adults and elderly subjects, and the effects of gender and age were assessed by measuring gait velocities, stride lengths, gait frequencies, and patterns of vertical acceleration signals during gait [16]. Vertical acceleration signal is a key feature in the evaluation of step symmetry and step regularity. These studies measured the acceleration and angular velocity signals that were generated by walking on the body center of gravity, legs, ankles, and feet, and separated the measured signals into steps to determine the abnormal gait pattern. In the study, walking was usually analyzed by the shape of the repeated waveform, peak, or frequency components [15,17,18,19]. The results were very similar to the results derived from the three-dimensional (3D) motion analyzer [20]. However, the acceleration and the angular velocity signals that were generated during walking are greatly influenced by individual walking characteristics, and even when diagnosed with hemiplegia, the waveform morphology varies greatly depending on the severity and age. Therefore, as in the previous study, the detection of abnormal gait via precise analysis of the waveform of each step requires substantial effort that is based on the detection process of the step and is limited by large-scale analytical errors associated with inaccurate step detection. More robust classification based on effective attributes are required to determine uncontrolled daily environments. In recent years, research is being conducted to classify gait patterns using accelerometer and pattern recognition techniques. In previous researches, classifier based on hidden Markov model and on support vector machine shows 90.5% accuracy with 90 probability and spatiotemporal features of inertial signal in distinguishing gait of the elderly, post-stroke patient and subjects with Huntington’s disease [21].

This study aims to simplify the characteristics of the acceleration signal that was measured during walking without step detection, instead of the current method that is associated with a high probability of false detection, to distinguish hemiplegic walking pattern. Particularly, this study intends to improve the accuracy of hemiplegic gait classification using inertial signals and pattern classification techniques.

## 2. Methods

A wearable measurement system that was equipped with an inertial sensor and wireless communication module was designed to measure and to distinguish hemiplegic gait from normal gait. Gait classification was based on attributes extracted from inertial signals of wearable sensing module without step detection. The type of walking was classified according to pattern recognition, and random forest (RF) algorithm. A forward search algorithm was used to determine the optimal classification attributes among the selected items. The proposed method was verified clinically.

### 2.1. Participants

The study was approved by the institutional review board (IRB) of the St. Carrolo Hospital (Suncheon, Jeollanam-do, South Korea) and all of the subjects signed informed consent before the experiment (IRB No. SCH2016-130). The participants included 20 normal subjects (10 males and 10 females; mean age 52.6 ± 16.3 years) and 20 hemiplegic patients (13 males and seven females, mean age 63.2 ± 8.9 years). The normal subjects were selected among volunteers who obtained normal scores (grade 5) in all of the MMT measurements that were performed by the rehabilitation therapist. Hemiplegic subjects were selected from patients diagnosed with stroke, who underwent rehabilitation, who did not have orthopedic disease, had a clear consciousness level, were able to understand the experiment, and were able to walk more than 20 m in the flat area without the help of a walker or an assistant. Among hemiplegia patients, 7, 10, and three patients had left-side hemiplegia, right-side hemiplegia, and bilateral hemiplegia, respectively. Five patients under the age of 40 were excluded from the analysis, when considering the effect of age on gait patterns. Therefore, data analysis was performed with a total of 35 subject’s data. Subject’s profile is shown in Table 1.

### 2.2. Development of Wearable System

In this study, we developed a wearable system to measure the acceleration that is caused by gait and a PC application for data display and storage. The wearable system consists of an inertial sensor module (MPU9250; InvenSense, San Jose, CA, USA) that is equipped with a three-axis accelerometer and three-axis gyroscope, microcontroller (MSP430G2553; Texas Instruments, Dallas, TX, USA), Bluetooth module (FB155BC; Firmtech, Sungnam, South Korea), and a rechargeable Li-Pol battery. The system was assembled as a printed circuit board (PCB) to minimize its size, with an inertial sensor located in the center of the PCB to prevent directional or rotational error. Specifications of MPU9250, MSP430G2553, and FB155BC are presented in Table 2, Table 3 and Table 4, respectively. A three-axis magnetometer was included in MPU9250; however, the specification of magnetometer was omitted in Table 2 because it was not used in this study.

**Figure 1:**
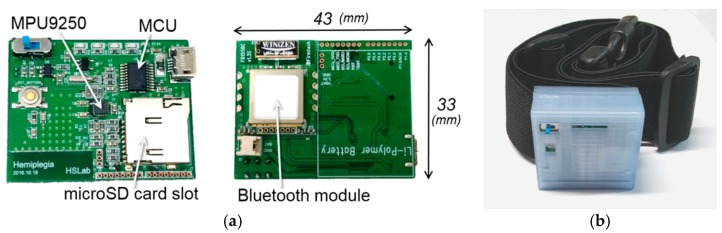
(**a**) Circuit board assembly and (**b**) developed system.

The circuit board measured 43 × 33 × 8.6 mm (W × H × D) in size, and the total system size, including cover was 47 × 44 × 20.5 mm (W × H × D). The system cover was created with free 3D modeling software (Autodesk 123D Design; Autodesk Inc., San-Rafael, CA, USA), and printed using a fused deposition modeling (FDM) (Makerbot Replicator 2; ABS filament; Stratasys Dimension 1200, ABS filament). The wearable system was worn on the body using a stretchable rubber belt (900 mm width × 38 mm in height). Figure 1a,b display the circuit board and the developed system, respectively.

### 2.3. Experimental Protocol

**Figure 2:**
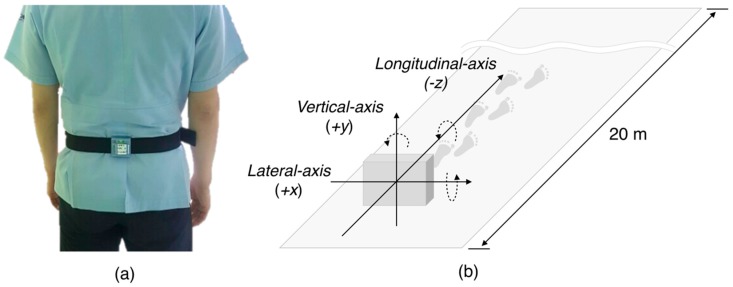
(**a**) Position of the wearable system between L3 and L4. (**b**) Direction of the wearable sensor. The solid line denotes the acceleration direction and the dashed line denotes angular velocity direction.

The wearable device was worn between lumbar 3 (L3) and 4 (L4), which is the body’s center of gravity (Figure 2a). The direction of acceleration was in the vertical (+*y*-axis), lateral (+*x*-axis), and longitudinal (−*z*-axis) axes (Figure 2b). The experiment was performed in a straight corridor lacking barriers to walking. The experimental procedure for data acquisition started with an explanation of the purpose and the experiment protocol to the candidate. Once informed consent was provided, the subjects wore the module on their waist and wait at the start line in a standing position. After the first start sign, each subject walked down the corridor for 20 m at a normal walking speed, and turned around to wait for the next start sign. After the second start sign, each subject walked back to the starting point at the same speed, which allowed two gait data per experiment, and 80 gait data were recorded in the 40 subjects.

### 2.4. Classification Attributes

In the case of hemiplegic walking, the center of gravity of the body deviates from the supporting plane. As a consequence, 60–80% of the body weight is based on the lower limb of the non-paralyzed side. Therefore, generally, the hemiplegic gait shows imbalance [22]. Furthermore, the gait of a hemiplegic patient is unstable compared with the normal gait due to muscle weakness and sensory degradation. To overcome unstable gait, abrupt compensation movement usually occurs. Compensation movement refers to a rapid movement in any direction, which generates large un-organized acceleration in every axis, contrary to the regular acceleration pattern that was observed in the normal gait.

Based on these characteristics of hemiplegic gait, we derived 165 attributes of hemiplegic and normal gait classification. The selected attributes include the average and maximum value of acceleration and angular rate in each axis, and the standard deviation of acceleration and angular rate in each axis that reflect irregular and unbalanced gait cycle. Moreover, attributes that may reflect gait frequency, regularity, and symmetry, such as kurtosis, skewness, and number of zero-crossing were also selected. Each attribute was not calculated for each step, but it was calculated using the 20 m walking signal as a whole. Details of the selected attributes are described in Table S1.

### 2.5. Attribute Selection

**Figure 3:**
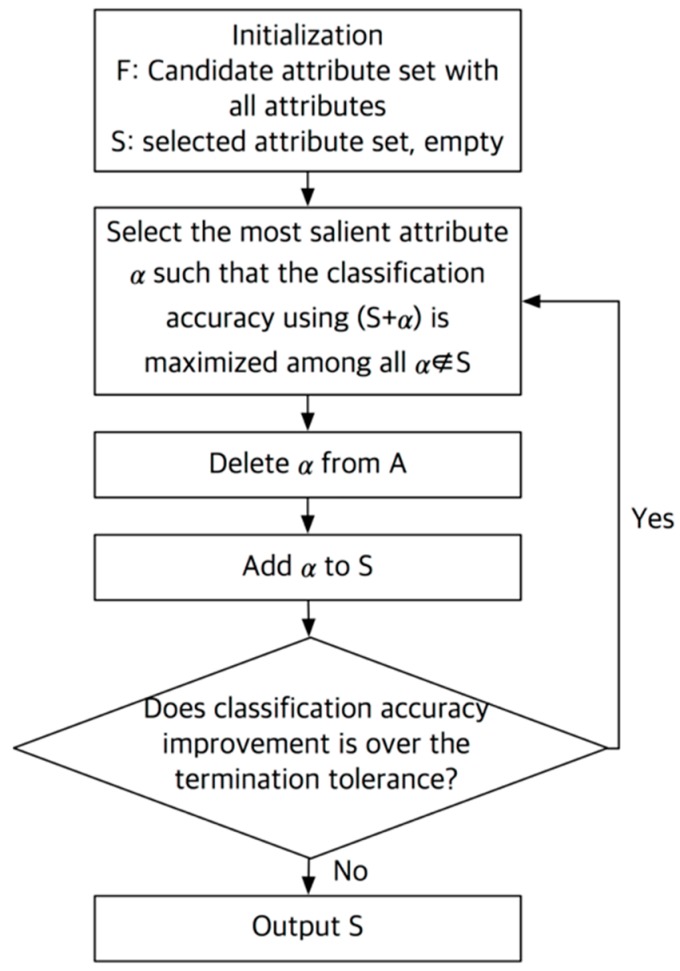
Flowchart of sequential forward search algorithm.

The accuracy of classification varies with the attribute set. Determination of the optimal combination of attributes is very important for the highest accuracy of classification. In this study, we used a sequential forward search algorithm for attribute selection [23], as outlined in Figure 3. In the sequential forward search algorithm, a set containing all attributes (*F*) and an empty set containing optimized attribute (*S*) were created at the initialization stage. Then, one attribute (*α*) was extracted from the *F* and was transferred to the *S*, and the classification using the *S* was repeated to select the *S* with the best classification accuracy or the lowest false classification rate. The process was reiterated until there was an improvement in accuracy or an error reduction based on a specific criterion, termination tolerance, as an additional attribute. As a result, the attribute set having the highest accuracy is found through a combination of specific attributes among the entire candidate attribute. In this study, we used random forest algorithm as a classifier, and the termination tolerance value was set to 10^−6^. In every procedure of extraction and selection of attributes with classification, we used MATLAB R2016a (Mathworks, Natick, MA, USA) and MATLAB function *sequentialfs* was used to implement sequential forward search algorithm.

### 2.6. Random Forest Classification

**Figure 4:**
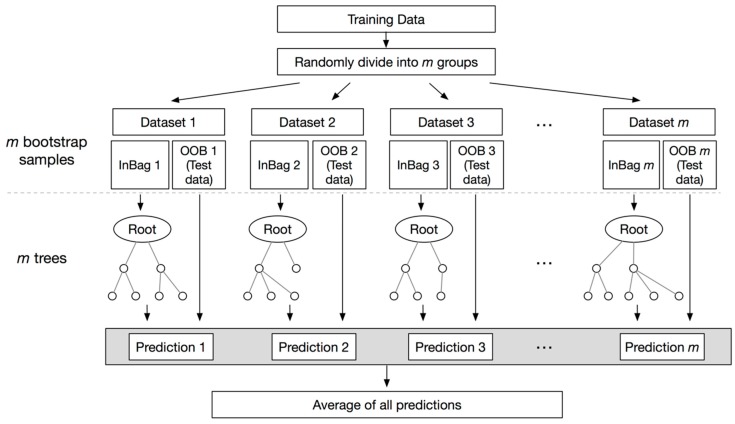
Flowchart of random forest algorithm.

RF is a regression technique that combines the performance of numerous decision tree algorithms to classify or to predict the value of a variable [24]. Input vector into the random forest leads to multiple regression trees and averages of the results. To avoid the correlation of the different trees, RF increases the diversity of the trees using different training datasets by bagging. Bagging is a technique used to create training data via random resampling of the original dataset with replacement. For example, bagging generates the next subset using independent random vectors with the same distribution without deletion of the data selected from the input sample. Therefore, a few data may be used more than once in the training, while others might never be used. This procedure renders the classifier more robust when facing slight variations in input data, and increases prediction accuracy [24]. Samples that are not selected in the bagging process are included as part of another subset, called out-of-bag (OOB). These OOB elements can be used by the *k*-th tree to evaluate performance [25]. Thus, RF computes an unbiased estimation of the generalization error without using an external text data subset [24]. Figure 4 shows the flowchart of the random forest regression. In this research, we produce a forest with a maximum of 50 trees. 

### 2.7. Statistical Analysis

We divided the data into 75% of training and verification sets (52 data) and 25% test sets (18 data) for gait classification using a random forest classifier. At the time, the ratio of normal and hemiplegic patients in each data set was kept constant, therefore, 30 of hemiplegic data and 26 of normal data, and 10 of hemiplegic data and eight of normal data were included in training set and in test set, respectively. In the training and validation stage, we used four-fold cross validation, which is a model validation technique to determine the generalization of the statistical analysis to an independent data set, and secure the reliability of the validation. The k-fold cross-validation is efficient in the absence of adequate data available for partition into separate training and test sets without losing significant modeling or testing capability. In four-fold cross-validation, the original dataset is randomly partitioned into four equal size sub-datasets. Of the four sub-datasets, a single sub-dataset is retained as the validation data for testing the model, and the remaining three sub-datasets are used as training data. The cross-validation process is then repeated four times, with each of the four sub-datasets being used exactly once as the validation data. Every result from the folds be averaged to produce a single estimation. The results were statistically analyzed with accuracy (*AC*), sensitivity (*SE*), specificity (*SP*), and positive predictive value (*PPV*). Here, *AC* is a probability of correct decision in every class, which can be interpreted in the same sense as the probability of detection of both normal and hemiplegic gait. *SE*, *SP*, and *PPV* are the abilities to correctly determine hemiplegic gait, the ability to determine normal gait correctly, and the percentage of hemiplegia patients with a positive test who actually manifest hemiplegia, respectively. Equations of measures are represented in Equations (1)–(4).

(1) $$Sensitivity\left(SE\right)=\frac{TP}{TP+FN}\times 100$$

(2) $$Specificity\left(SP\right)=\frac{TN}{FP+TN}\times 100$$

(3) $$Accuracy\left(AC\right)=\frac{TP+TN}{TP+FP++TN+FN}\times 100$$

(4) $$Positive\;Predictivity\;Value\left(PPV\right)=\frac{TP}{TP+FP}\times 100$$

## 3. Results

### 3.1. Signal Acquisition

**Figure 5:**
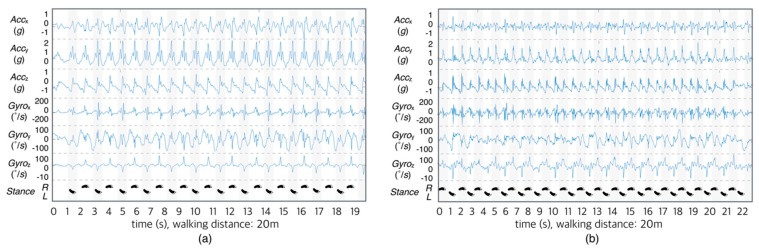
Signal acquisition during gait via lateral-axis acceleration, vertical-axis acceleration, longitudinal-axis acceleration, angular velocity around lateral-axis, angular velocity around vertical-axis and angular velocity around longitudinal-axis (from top). (**a**) a normal subject (subject No. 22) and (**b**) a hemiplegic patient (subject no. 1).

The signal that was acquired by the wearable system includes both the signal in the standby state and the signal in the walking state. In this study, the signals that are generated during non-reporting were used only for calibration and excluded from the analysis. Figure 5 shows an example of a measured signal with a normal subject (Figure 5a) and with a hemiplegic subject (Figure 5b) during the 20-m corridor walk. As shown in the figure, the acceleration and the angular velocity were recorded correctly. Moreover, we intuitively observed the difference between normal and hemiplegic gait. The normal gait displayed fewer steps and a neat waveform when compared with hemiplegic gait signal. In the case of normal walk, regular gait patterns were observed when walking bilaterally.

### 3.2. Classification Results

**Figure 6:**
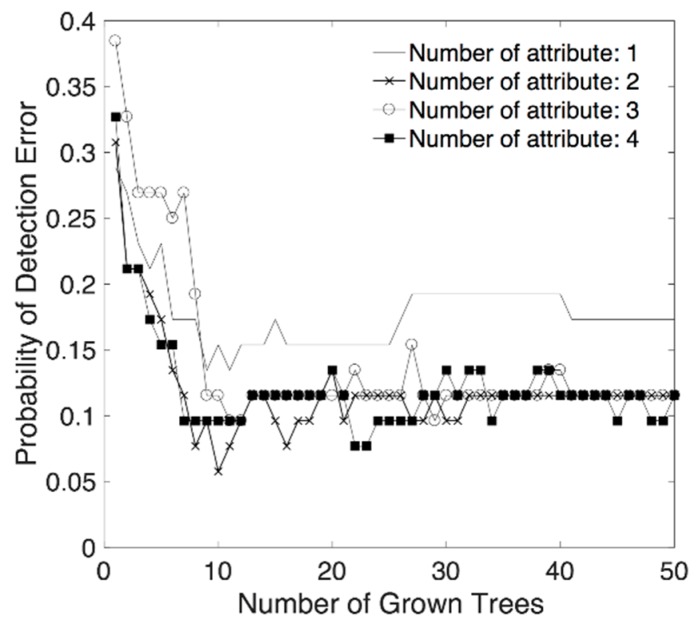
Probability of detection error of training set according to the number of classification attributes and the number of trees.

According to the sequential forward search algorithm with 10^−6^ termination tolerance, we obtained classification accuracy according to the combination of classification attributes and the number of trees. Changes of probability of detection error, in the training stage, according to the number of attributes and the number of trees are presented in Figure 6. This result shows that the detection error is reduced by growth of tree, however, no significant improvement in accuracy was observed when the number of trees exceeded around 10. For the number of classification attributes, no outstanding difference in accuracy was observed when the number of combined classification attributes exceeded two when the number of trees was 10 or more. 

**Figure 7:**
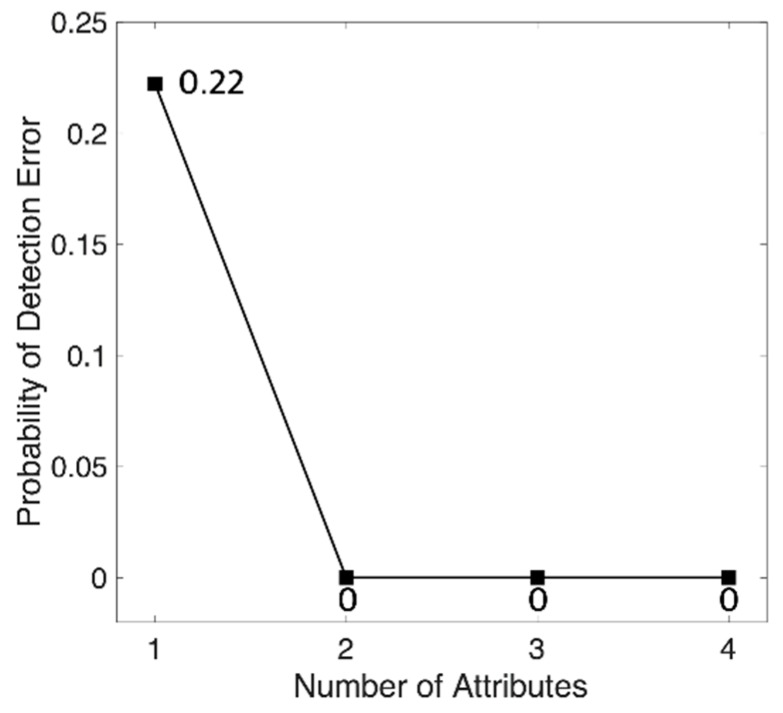
Probability of detection error of test set. Classification attributes were added in order of *SD1*_GYROY_, *Kur*_GYROZ_, *Skew*_GYROZ_, and *SDZCIL*_ACCZ_. Number of tree is 50.

We found an optimal set of attributes using a sequential forward search from a randomized training set. At this time, the number of optimal attributes varied from 2 to 4, according to the training set, and the most frequently selected attributes after 100 iterations were determined as optimal attributes. Top four classification attribute that were evaluated as having the highest classification contribution by sequential forward search are the standard deviation of points perpendicular to the axis of line of identity of Poincaré plot of angular velocity around vertical axis (*SD1*_GYROY_), kurtosis of frequency of angular velocity around longitudinal axis (*Kur*_GYROZ_), skewness of frequency of angular velocity around longitudinal axis (*Skew*_GYROZ_), and standard deviation of time interval of adjacent local maxima of acceleration on longitudinal axis (*SDZCIL*_ACCZ_). For a test, we generated forest using 1 to 4 selected attributed, and then calculated the classification accuracy that was based on test set. Figure 7 shows the probability of detection error of classification. The result of test shows that the probability of classification error is 0 when we use 2 or more classification attributes with 50 trees. Therefore, we finally selected two classification attributes that were evaluated as having the highest classification contribution by sequential forward search. Selected attributes are the standard deviation of points perpendicular to the axis of line of identity of Poincaré plot of angular velocity around vertical axis (*SD1*_GYROY_) and kurtosis of frequency of angular velocity around longitudinal axis (*Kur*_GYROZ_).

Table 5 shows the confusion matrix as a result of the classification using test set. In the case of normal gait, eight out of eight normal gait data sets were classified as normal gait and a 100.0% of *AC* was obtained. In the case of hemiplegic gait, 10 out of 10 hemiplegic gait data sets were correctly classified (100.0% of *AC*). The total *AC* for both normal and hemiplegia was 100.0% (18 of 18), and *SE*, *SP*, and *PPV* were 100.0%, 100.0%, and 100.0%, respectively. This test result suggests that at least two features—*SD1*_GYROY_ and *Kur*_GYROZ_—are required for classifying hemiplegic gait reliably for a given data set. Robustness might be improved by adding proper other features.

## 4. Discussion

A simple characteristic of the acceleration signal that is caused by asymmetry in walking can be used to classify hemiplegic gait. In the case of hemiplegic patients, the left and the right sides are not in equilibrium and the paralyzed side is unstable because of excessive reflexes, muscle spasms, muscular resistance, and muscular rigidity. Therefore, several body movements occur in the left and right axis during walking [16], which are effective in distinguishing the presence of hemiplegia. Furthermore, in patients with hemiplegia, a discontinuous gait is caused by an increase in the bilateral support period [26]. 

Among the selected classification attributes, *SD1*_GYROY_, as the acceleration or the rotational angular velocity signal, abruptly changes due to the nature of the Poincaré plot; the *SD1* value becomes larger because the data is distributed at a distance from the identity function. In patients with hemiplegia, asymmetry of the left- and right-side is higher than that of the normal subjects, and the change of the angular velocity signal is sharpened, so it is expected that the value of *SD1*_GYROY_ is different. The classification attribute *Kur*_GYROZ_, which indicates the center frequency kurtosis of the rotational angular velocity around the longitudinal-axis, has a moderate kurtosis when compared with the normal person distinguishing the paralyzed gait based on differences in stride or lifting period [5,26]. Therefore, it is presumed that this attribute reflects the difference between hemiplegic and normal gait patterns.

## 5. Conclusions

The present classification of hemiplegic gait using wearable devices was based on simple characteristics of the acceleration signal that is caused by asymmetric gait. The proposed technique provides a rough screening tool for the detection of hemiparesis. The proposed method is only based on raw inertial signal that is measured during walking. In other words, it can be applied without preprocessing with a high degree of error probability, such as step detection. In terms of attribute, *SD1*_GYROY_ and *Kur*_GYROZ_ is a minimal attribute set for optimal result. However, the accuracy can be increased by adding a classification attribute with high significance, which reflects the reliable operation characteristics and future patterns of hemiplegic walking. Acquisition and analysis of signals over an extended period of time are also necessary to monitor the progress and the recovery of hemiplegic gait. Therefore, long-term follow-up studies are needed to determine the severity of hemiplegia. There are some limitations of this study due to the population. Since this study is not based on Big Data, we did not apply the more advanced classification technique, such as Deep Learning. If we accumulate data continuously, we can get improved results by applying deep running and so on. Moreover, this study is only designed for older (>40 years) subjects. Therefore, in order to obtain more reliable results, extended experimentation and evaluation should be conducted for a large number of subjects with various age, sex, and symptom severity. Further, for more general use, it is necessary to be considered to measurement technology independent of the mounting position of the system.

## Figures and Tables

**Table 1:** Subject’s profile.

Hemiplegia						Normal				
Subject No.	Paralyzed Side (L/R/B)	Gender (M/F)	Age (years)	Height (cm)	Weight (kg)	Subject No.	Gender (M/F)	Age (years)	Height (cm)	Weight (kg)
1	L	M	54	174	80	21	M	48	168	63
2	R	M	65	173	74	22	M	45	167	65
3	R	M	76	170	90	23	M	44	170	75
4	L	M	59	167	73	24	F	57	159	54
5	R	M	72	177	75	25	F	68	150	51
6	R	M	63	172	70	26	F	58	163	80
7	L	M	75	175	70	27	M	71	165	68
8	B	M	63	176	64	28	F	71	158	62
9	B	F	64	150	45	29	F	71	157	68
10	R	M	60	178	79	30	F	64	156	54
11	R	M	78	170	67	31	M	77	168	80
12	R	F	63	163	67	32	F	69	155	66
13	L	F	60	150	38	33	F	64	162	72
14	L	M	68	168	58	34	M	46	171	82
15	L	F	64	152	50	35	M	42	173	69
16	B	M	47	175	75					
17	R	F	49	162	90					
18	L	F	73	145	50					
19	R	F	62	150	45					
20	R	M	49	171	75					
Mean ± SD	L: 7, R: 10, B: 3	M: 13, F: 7	63.2 ± 8.9	165.9 ± 10.7	66.8 ± 14.8	Mean ± SD	M: 7, F: 8	59.7 ± 11.9	162.8 ± 6.8	67.3 ± 9.6

**Table 2:** Specification of the accelerometer and gyroscope in MPU9250.

Type	Feature	Value
Accelerometer	Full-scale range	±16 g
	ADC word length	16 bits
	Sensitivity scale factor	16,384 LSB/g
	Sensitivity change vs. temperature	±0.026%/°C
	Cross-axis sensitivity	±2%
	Noise power spectral density	300 µg/√Hz
	Total RMS noise	8 mg-rms
	Maximum output data rate	4000 Hz
Gyroscope	Full-scale range	±2000°/s
	ADC word length	16 bits
	Sensitivity scale factor	131 LSB/(°/s)
	Sensitivity scale factor variation over temperature	±4%
	Cross-axis sensitivity	±2%
	Total RMS noise	0.1°/s-rms
	Rate noise spectral density	0.01°/s/√Hz
	Maximum output data rate	8000 Hz
Communication	I^2^C operating frequency	400 kHz (Fast-mode) 100 kHz (Standard-mode)

**Table 3:** Specification of the microcontroller unit, MSP430G2553.

Feature	Description
Supply-voltage range	1.8 V to 3.6 V
Power consumption	Active Mode: 230 µA at 1 MHz, 2.2 V Standby Mode: 0.5 µA Off Mode (RAM Retention): 0.1 µA
Analog-to-digital (A/D) converter	10-Bit 200-ksps
Universal Serial Communication Interface (USCI)	Enhanced UART Supporting Auto Baud Rate Detection (LIN) IrDA Encoder and Decoder Synchronous SPI I^2^C
Wake-up time from standby mode	<1 us
Frequency	up to 16 MHz
Timer	2 16-Bit Timer_A With 3 Capture/Compare Registers

**Table 4:** Specification of the Bluetooth module, FB155BC.

Feature	Description
Power Class	Class2
RF Range	Up to 30 m
Power Voltage	DC 3.3 V
Serial Interface	UART
Flow Control	RTS, CTS Support
Bluetooth Profile	Serial Port Profile
Bluetooth Version	1.2
Applicable Antenna	Included Chip Antenna
Certification	MIC
Dimension	18 × 20 mm

**Table 5:** Confusion matrix of the classification using test set.

		Estimated	
		Hemiplegic Gait	Normal Gait
Actual	hemiplegic gait	10	0
	normal gait	0	8

## Supplementary Materials

The following is available online at http://www.mdpi.com/1424-8220/18/6/1736/s1, Table S1: List of attribute candidates.
