# Supplementary material for: Detection of Hemiplegic Walking Using a Wearable Inertia Sensing Device
Source: Sensors (Basel). 2018 May 28;18(6):1736. doi: 10.3390/s18061736 (PMC6022123; doi:10.3390/s18061736)
Supplement: Supplementary file 1 [file sensors-18-01736-s001.pdf]

Supplementary Material

Table S1. List of attribute candidates

**No.**	**Attribute Name**	**Description**
1	Avg_{ACCX}	Average of acceleration on lateral axis
2	Avg_{ACCY}	Average of acceleration on vertical axis
3	Avg_{ACCZ}	Average of acceleration on longitudinal axis
4	Avg_{GYROX}	Average of angular velocity around lateral axis
5	Avg_{GYROY}	Average of angular velocity around vertical axis
6	Avg_{GYROZ}	Average of angular velocity around longitudinal axis
7	SD_{ACCX}	Standard deviation of acceleration on lateral axis
8	SD_{ACCY}	Standard deviation of acceleration on vertical axis
9	SD_{ACCZ}	Standard deviation of acceleration on longitudinal axis
10	SD_{GYROX}	Standard deviation of angular velocity around lateral axis
11	SD_{GYROY}	Standard deviation of angular velocity around vertical axis
12	SD_{GYROZ}	Standard deviation of angular velocity around longitudinal axis
13	NZC_{ACCX}	Number of zero-crossing of acceleration on lateral axis
14	NZC_{ACCY}	Number of zero-crossing of acceleration on vertical axis
15	NZC_{ACCZ}	Number of zero-crossing of acceleration on longitudinal axis
16	NZC_{GYROX}	Number of zero-crossing of angular velocity around lateral axis
17	NZC_{GYROY}	Number of zero-crossing of angular velocity around vertical axis
18	NZC_{GYROZ}	Number of zero-crossing of angular velocity around longitudinal axis
19	$AvgZCI_{\mathrm{ACCX}}$	Average of zero-crossing interval of acceleration on lateral axis
20	$AvgZCI_{\mathrm{ACCY}}$	Average of zero-crossing interval of acceleration on vertical axis
21	$AvgZCI_{\mathrm{ACCZ}}$	Average of zero-crossing interval of acceleration on longitudinal axis
22	$AvgZCI_{\mathrm{GYROX}}$	Average of zero-crossing interval of angular velocity around lateral axis
23	$AvgZCI_{\mathrm{GYROY}}$	Average of zero-crossing interval of angular velocity around vertical axis
24	$AvgZCI_{\mathrm{GYROZ}}$	Average of zero-crossing interval of angular velocity around longitudinal axis
25	$SDZCI_{\mathrm{ACCX}}$	Standard deviation of zero-crossing interval of acceleration on lateral axis
26	$SDZCI_{\mathrm{ACCY}}$	Standard deviation of zero-crossing interval of acceleration on vertical axis
27	$SDZCI_{\mathrm{ACCZ}}$	Standard deviation of zero-crossing interval of acceleration on longitudinal axis
28	$SDZCI_{\mathrm{GYROX}}$	Standard deviation of zero-crossing interval of angular velocity around lateral axis
29	$SDZCI_{\mathrm{GYROY}}$	Standard deviation of zero-crossing interval of angular velocity around vertical axis
30	$SDZCI_{\mathrm{GYROZ}}$	Standard deviation of zero-crossing interval of angular velocity around longitudinal axis
31	$SDZCIU_{\mathrm{ACCX}}$	Standard deviation of time interval of adjacent local maxima of acceleration on lateral axis
32	$SDZCIU_{\mathrm{ACCY}}$	Standard deviation of time interval of adjacent local maxima of acceleration on vertical axis

33	$SDZCIU_{\mathrm{ACCZ}}$	Standard deviation of time interval of adjacent local maxima of acceleration on longitudinal axis
34	$SDZCIU_{\mathrm{GYROX}}$	Standard deviation of time interval of adjacent local maxima of angular velocity around lateral axis
35	$SDZCIU_{\mathrm{GYROY}}$	Standard deviation of time interval of adjacent local maxima of angular velocity around vertical axis
36	$SDZCIU_{\mathrm{GYROZ}}$	Standard deviation of time interval of adjacent local maxima of angular velocity around longitudinal axis
37	$SDZCIL_{\mathrm{ACCX}}$	Standard deviation of time interval of adjacent local minima of acceleration on lateral axis
38	$SDZCIL_{\mathrm{ACCY}}$	Standard deviation of time interval of adjacent local maxima of acceleration on vertical axis
39	$SDZCIL_{\mathrm{ACCZ}}$	Standard deviation of time interval of adjacent local maxima of acceleration on longitudinal axis
40	$SDZCIL_{\mathrm{GYROX}}$	Standard deviation of time interval of adjacent local maxima of angular velocity around lateral axis
41	$SDZCIL_{\mathrm{GYROY}}$	Standard deviation of time interval of adjacent local maxima of angular velocity around vertical axis
42	$SDZCIL_{\mathrm{GYROZ}}$	Standard deviation of time interval of adjacent local maxima of angular velocity around longitudinal axis
43	$SD1_{\mathrm{ACCX}}$	Standard deviation of points perpendicular to the axis of line of identity of Poincaré plot of acceleration on lateral axis
44	$SD1_{\mathrm{ACCY}}$	Standard deviation of points perpendicular to the axis of line of identity of Poincaré plot of acceleration on vertical axis
45	$SD1_{\mathrm{ACCZ}}$	Standard deviation of points perpendicular to the axis of line of identity of Poincaré plot of acceleration on longitudinal axis
46	$SD1_{\mathrm{GYROX}}$	Standard deviation of points perpendicular to the axis of line of identity of Poincaré plot of angular velocity around lateral axis
47	$SD1_{\mathrm{GYROY}}$	Standard deviation of points perpendicular to the axis of line of identity of Poincaré plot of angular velocity around vertical axis
48	$SD1_{\mathrm{GYROZ}}$	Standard deviation of points perpendicular to the axis of line of identity of Poincaré plot of angular velocity around longitudinal axis
49	$SD2_{\mathrm{ACCX}}$	Standard deviation of points along the axis of line of identity of Poincaré plot of acceleration on lateral axis
50	$SD2_{\mathrm{ACCY}}$	Standard deviation of points along the axis of line of identity of Poincaré plot of acceleration on vertical axis
51	$SD2_{\mathrm{ACCZ}}$	Standard deviation of points along the axis of line of identity of Poincaré plot of acceleration on longitudinal axis
52	$SD2_{\mathrm{GYROX}}$	Standard deviation of points along the axis of line of identity of Poincaré plot of angular velocity around lateral axis
53	$SD2_{\mathrm{GYROY}}$	Standard deviation of points along the axis of line of identity of Poincaré plot of angular velocity around vertical axis
54	$SD2_{\mathrm{GYROZ}}$	Standard deviation of points along the axis of line of identity of Poincaré plot of angular velocity around longitudinal axis
55	$FMAX_{\mathrm{ACCX}}$	Maximum value of frequency of acceleration on lateral axis
56	$FMAX_{\mathrm{ACCY}}$	Maximum value of frequency of acceleration on vertical axis
57	$FMAX_{\mathrm{ACCZ}}$	Maximum value of frequency of acceleration on longitudinal axis
58	$FMAX_{\mathrm{GYROX}}$	Maximum value of frequency of angular velocity around lateral axis
59	$FMAX_{\mathrm{GYROY}}$	Maximum value of frequency of angular velocity around vertical axis

60	$FMAX_{\mathrm{GYROZ}}$	Maximum value of frequency of angular velocity around longitudinal axis
61	Kur_{ACCX}	Kurtosis of frequency of acceleration on lateral axis
62	Kur_{ACCY}	Kurtosis of frequency of acceleration on vertical axis
63	Kur_{ACCZ}	Kurtosis of frequency of acceleration on longitudinal axis
64	Kur_{GYROX}	Kurtosis of frequency of angular velocity around lateral axis
65	Kur_{GYROY}	Kurtosis of frequency of angular velocity around vertical axis
66	Kur_{GYROZ}	Kurtosis of frequency of angular velocity around longitudinal axis
67	$Skew_{\mathrm{ACCX}}$	Skewness of frequency of acceleration on lateral axis
68	$Skew_{\mathrm{ACCY}}$	Skewness of frequency of acceleration on vertical axis
69	$Skew_{\mathrm{ACCZ}}$	Skewness of frequency of acceleration on longitudinal axis
70	$Skew_{\mathrm{GYROX}}$	Skewness of frequency of angular velocity around lateral axis
71	$Skew_{\mathrm{GYROY}}$	Skewness of frequency of angular velocity around vertical axis
72	$Skew_{\mathrm{GYROZ}}$	Skewness of frequency of angular velocity around longitudinal axis
73	MAX_{ACCX}	Maximum value of acceleration on lateral axis
74	MAX_{ACCY}	Maximum value of acceleration on vertical axis
75	MAX_{ACCZ}	Maximum value of acceleration on longitudinal axis
76	MAX_{GYROX}	Maximum value of angular velocity around lateral axis
77	MAX_{GYROY}	Maximum value of angular velocity around vertical axis
78	MAX_{GYROZ}	Maximum value of angular velocity around longitudinal axis
79	Avg_{ACCXY}	Average of acceleration ratio of the lateral axis to the vertical axis
80	Avg_{ACCXZ}	Average of acceleration ratio of the lateral axis to the longitudinal axis
81	Avg_{ACCYZ}	Average of acceleration ratio of the vertical axis to the longitudinal axis
82	Avg_{GYROXY}	Average of angular velocity ratio of the lateral axis to the vertical axis
83	Avg_{GYROXZ}	Average of angular velocity ratio of the lateral axis to the vertical axis
84	Avg_{GYROYZ}	Average of angular velocity ratio of the vertical axis to the longitudinal axis
85	SD_{ACCXY}	Standard deviation of acceleration ratio of the lateral axis to the vertical axis
86	SD_{ACCXZ}	Standard deviation of acceleration ratio of the lateral axis to the longitudinal axis
87	SD_{ACCYZ}	Standard deviation of acceleration ratio of the vertical axis to the longitudinal axis
88	SD_{GYROXY}	Standard deviation of angular velocity ratio of the lateral axis to the vertical axis
89	SD_{GYROXZ}	Standard deviation of angular velocity ratio of the lateral axis to the vertical axis
90	SD_{GYROYZ}	Standard deviation of angular velocity ratio of the vertical axis to the longitudinal axis
91	NZC_{ACCXY}	Number of zero-crossing of acceleration ratio of the lateral axis to the vertical axis
92	NZC_{ACCXZ}	Number of zero-crossing of acceleration ratio of the lateral axis to the longitudinal axis
93	NZC_{ACCYZ}	Number of zero-crossing of acceleration ratio of the vertical axis to the longitudinal axis
94	NZC_{GYROXY}	Number of zero-crossing of angular velocity ratio of the lateral axis to the vertical axis
95	NZC_{GYROXZ}	Number of zero-crossing of angular velocity ratio of the lateral axis to the vertical axis

96	NZC_{GYROYZ}	Number of zero-crossing of angular velocity ratio of the vertical axis to the longitudinal axis
97	$AvgZCI_{\mathrm{ACCXY}}$	Average of zero-crossing interval of acceleration ratio of the lateral axis to the vertical axis
98	$AvgZCI_{\mathrm{ACCXZ}}$	Average of zero-crossing interval of acceleration ratio of the lateral axis to the longitudinal axis
99	$AvgZCI_{ACCYZ}$	Average of zero-crossing interval of acceleration ratio of the vertical axis to the longitudinal axis
100	$AvgZCI_{\mathrm{GYROXY}}$	Average of zero-crossing interval of angular velocity ratio of the lateral axis to the vertical axis
101	$AvgZCI_{\mathrm{GYROXZ}}$	Average of zero-crossing interval of angular velocity ratio of the lateral axis to the vertical axis
102	$AvgZCI_{\mathrm{GYROYZ}}$	Average of zero-crossing interval of angular velocity ratio of the vertical axis to the longitudinal axis
103	$SDZCI_{\mathrm{ACCXY}}$	Standard deviation of zero-crossing interval of acceleration ratio of the lateral axis to the vertical axis
104	$SDZCI_{\mathrm{ACCXZ}}$	Standard deviation of zero-crossing interval of acceleration ratio of the lateral axis to the longitudinal axis
105	$SDZCI_{\mathrm{ACCYZ}}$	Standard deviation of zero-crossing interval of acceleration ratio of the vertical axis to the longitudinal axis
106	$SDZCI_{\mathrm{GYROXY}}$	Standard deviation of zero-crossing interval of angular velocity ratio of the lateral axis to the vertical axis
107	$SDZCI_{\mathrm{GYROXZ}}$	Standard deviation of zero-crossing interval of angular velocity ratio of the lateral axis to the vertical axis
108	$SDZCI_{\mathrm{GYROYZ}}$	Standard deviation of zero-crossing interval of angular velocity ratio of the vertical axis to the longitudinal axis
109	$SDZCIU_{\mathrm{ACCXY}}$	Standard deviation of time interval ratio of adjacent local maxima of acceleration of the lateral axis to the vertical axis
110	$SDZCIU_{\mathrm{ACCXZ}}$	Standard deviation of time interval ratio of adjacent local maxima of acceleration of the lateral axis to the vertical axis
111	$SDZCIU_{\mathrm{ACCYZ}}$	Standard deviation of time interval ratio of adjacent local maxima of acceleration of the vertical axis to the longitudinal axis
112	$SDZCIU_{\mathrm{GYROXY}}$	Standard deviation of time interval ratio of adjacent local maxima of angular velocity of lateral axis to the vertical axis
113	$SDZCIU_{\mathrm{GYROXZ}}$	Standard deviation of time interval ratio of adjacent local maxima of angular velocity of lateral axis to the vertical axis
114	$SDZCIU_{\mathrm{GYROYZ}}$	Standard deviation of time interval ratio of adjacent local maxima of angular velocity of vertical axis to the longitudinal axis
115	$SDZCIL_{\mathrm{ACCXY}}$	Standard deviation of time interval ratio of adjacent local minima of acceleration of the lateral axis to the vertical axis
116	$SDZCIL_{\mathrm{ACCXZ}}$	Standard deviation of time interval ratio of adjacent local minima of acceleration of the lateral axis to the vertical axis
117	$SDZCIL_{\mathrm{ACCYZ}}$	Standard deviation of time interval ratio of adjacent local minima of acceleration of the vertical axis to the longitudinal axis
118	$SDZCIL_{\mathrm{GYROXY}}$	Standard deviation of time interval ratio of adjacent local minima of angular velocity of lateral axis to the vertical axis
119	$SDZCIL_{\mathrm{GYROXZ}}$	Standard deviation of time interval ratio of adjacent local minima of angular velocity of lateral axis to the vertical axis

120	$SDZCIL_{\mathrm{GYROYZ}}$	Standard deviation of time interval ratio of adjacent local minima of angular velocity of vertical axis to the longitudinal axis
121	$SD1_{\mathrm{ACCXY}}$	Standard deviation of points perpendicular to the axis of line of identity of Poincaré plot of acceleration ratio on the lateral axis to the vertical axis
122	$SD1_{\mathrm{ACCXZ}}$	Standard deviation of points perpendicular to the axis of line of identity of Poincaré plot of acceleration ratio on the lateral axis to the longitudinal axis
123	$SD1_{\mathrm{ACCYZ}}$	Standard deviation of points perpendicular to the axis of line of identity of Poincaré plot of acceleration ratio on the vertical axis to the longitudinal axis
124	$SD1_{\mathrm{GYROXY}}$	Standard deviation of points perpendicular to the axis of line of identity of Poincaré plot of angular velocity ratio around lateral axis to the vertical axis
125	$SD1_{\mathrm{GYROXZ}}$	Standard deviation of points perpendicular to the axis of line of identity of Poincaré plot of angular velocity ratio around lateral axis to the longitudinal axis
126	$SD1_{\mathrm{GYROYZ}}$	Standard deviation of points perpendicular to the axis of line of identity of Poincaré plot of angular velocity ratio around vertical to the longitudinal axis
127	$SD2_{\mathrm{ACCXY}}$	Standard deviation of points along to the axis of line of identity of Poincaré plot of acceleration ratio on the lateral axis to the vertical axis
128	$SD2_{\mathrm{ACCXZ}}$	Standard deviation of points along to the axis of line of identity of Poincaré plot of acceleration ratio on the lateral axis to the longitudinal axis
129	$SD2_{\mathrm{ACCYZ}}$	Standard deviation of points along to the axis of line of identity of Poincaré plot of acceleration ratio on the vertical axis to the longitudinal axis
130	$SD2_{\mathrm{GYROXY}}$	Standard deviation of points along to the axis of line of identity of Poincaré plot of angular velocity ratio around lateral axis to the vertical axis
131	$SD2_{\mathrm{GYROXZ}}$	Standard deviation of points along to the axis of line of identity of Poincaré plot of angular velocity ratio around lateral axis to the longitudinal axis
132	$SD2_{\mathrm{GYROYZ}}$	Standard deviation of points along to the axis of line of identity of Poincaré plot of angular velocity ratio around vertical to the longitudinal axis
133	$FMAX_{\mathrm{ACCXY}}$	Maximum value of frequency of acceleration ratio of lateral axis to the vertical axis
134	$FMAX_{\mathrm{ACCXZ}}$	Maximum value of frequency of acceleration ratio of lateral axis to the longitudinal axis
135	$FMAX_{\mathrm{ACCYZ}}$	Maximum value of frequency of acceleration ratio of the vertical axis to the longitudinal axis
136	$FMAX_{\mathrm{GYROXY}}$	Maximum value of frequency of angular velocity ratio around lateral axis to the vertical axis
137	$FMAX_{\mathrm{GYROXZ}}$	Maximum value of frequency of angular velocity ratio around the lateral axis to the vertical axis
138	$FMAX_{\mathrm{GYROYZ}}$	Maximum value of frequency of angular velocity ratio around the vertical axis to the longitudinal axis
139	Kur_{ACCXY}	Kurtosis of frequency of acceleration ratio of the lateral axis to the vertical axis
140	Kur_{ACCXZ}	Kurtosis of frequency of acceleration ratio of the lateral axis to the longitudinal axis
141	Kur_{ACCYZ}	Kurtosis of frequency of acceleration ratio of the vertical axis to the longitudinal axis
142	Kur_{GYROXY}	Kurtosis of frequency of angular velocity ratio around the lateral axis to the vertical axis
143	Kur_{GYROXZ}	Kurtosis of frequency of angular velocity ratio around the lateral axis to the longitudinal axis

144	Kur_{GYROYZ}	Kurtosis of frequency of angular velocity ratio around the vertical axis to the longitudinal axis
145	$Skew_{\mathrm{ACCXY}}$	Skewness of frequency of acceleration ratio of the lateral axis to the vertical axis
146	$Skew_{\mathrm{ACCXZ}}$	Skewness of frequency of acceleration ratio of the lateral axis to the longitudinal axis
147	$Skew_{\mathrm{ACCYZ}}$	Skewness of frequency of acceleration ratio of the vertical axis to the longitudinal axis
148	$Skew_{\mathrm{GYROXY}}$	Skewness of frequency of angular velocity ratio around the lateral axis to the vertical axis
149	$Skew_{\mathrm{GYROXZ}}$	Skewness of frequency of angular velocity ratio around the lateral axis to the longitudinal axis
150	$Skew_{\mathrm{GYROYZ}}$	Skewness of frequency of angular velocity ratio around the vertical axis to the longitudinal axis
151	$AvgSum_{\mathrm{ACCXY}}$	Average value of lateral axis and vertical axis acceleration
152	$AvgSum_{\mathrm{ACCXZ}}$	Average value of lateral axis and longitudinal axis acceleration
153	$AvgSum_{\mathrm{ACCYZ}}$	Average value of vertical axis and longitudinal axis acceleration
154	$AvgSum_{\mathrm{ACCXYZ}}$	Average value of angular velocity around lateral axis, vertical axis and longitudinal axis
155	$AvgDifLR_{\mathrm{ACCX}}$	Average difference of acceleration on lateral axis when subject stamps on the ground
156	$AvgDifLR_{\mathrm{ACCYZ}}$	Average difference of acceleration of the vertical axis to the longitudinal axis when subject stamps on the ground
157	$AvgDifLR_{\mathrm{GYROX}}$	Average difference of angular velocity of vertical axis around to the lateral axis when subject stamps on the ground
158	$AvgDifLRG_{\mathrm{ACCX}}$	Average difference of acceleration between left and right on lateral axis
159	$AvgDifLRG_{\mathrm{ACCY}}$	Average difference of acceleration between left and right on vertical axis
160	$AvgDifLRG_{\mathrm{ACCZ}}$	Average difference of acceleration between left and right on longitudinal axis
161	$AvgDifLRG_{\mathrm{GYROY}}$	Average difference between left and right angular velocity of vertical axis around to the lateral axis
162	$AvgSumF_{\mathrm{ACCXZ}}$	An average of the sum of the lateral axis acceleration and the longitudinal axis acceleration when forward acceleration is generated
163	$VarR_{\mathrm{ACCX}}$	Variance of lateral axis acceleration when an acceleration signal is applied to the rear during walking
164	$VarR_{\mathrm{ACCY}}$	Variance of vertical axis acceleration when an acceleration signal is applied to the rear during walking
165	$VarR_{\mathrm{GYROZ}}$	Variance of angular velocity around longitudinal axis when an acceleration signal is applied to the rear during walking
